# Mental Health, Bullying, and Victimization among Chinese Adolescents

**DOI:** 10.3390/children9020240

**Published:** 2022-02-11

**Authors:** Yang Wen, Xihe Zhu, Justin A. Haegele, Fangliang Yu

**Affiliations:** 1 College of Sports Industry and Leisure, Nanjing Sport Institute, Nanjing 210014, China; 2001070012@nsi.edu.cn; 2 Department of Human Movement Sciences, Old Dominion University, Norfolk, VA 23508, USA; x2zhu@odu.edu (X.Z.); jhaegele@odu.edu (J.A.H.); 3 School of Sports Training, Nanjing Sport Institute, Nanjing 210014, China

**Keywords:** anxiety, depression, bully, victim, gender, student

## Abstract

The purpose of this study was to examine if adolescents who experience anxiety or depression have higher levels of reported bullying victimization or perpetration than those who do not. Based on the existing research, we hypothesized that those who experienced moderate or severe depression and anxiety would have higher rates of bullying victimization and perpetration when compared to those who experienced mild or no depression. This study used an observational design, and data were collected from a convenience sample of adolescents in a large regional high school in an Eastern province of China. The final sample included 1481 adolescents aged 14–19 years who provided complete data for each of the study variables. Demographic data were collected through a four-item demographic survey, bullying perpetration and victimization data were collected using subscales from the Illinois Bully Scale, and anxiety and depression were measured using the Chinese version of the General Anxiety Disorder-7 scale and the Patient Health Questionnaire, respectively. Descriptive analyses, correlational analyses, and multivariate analysis of covariance were used to analyze the data. About 7.1% and 15.2% of participants reported moderate-to-severe depression or moderate-to-severe anxiety, respectively. Pairwise comparisons indicated that adolescents with no or mild depression had significantly lower bullying perpetration than those with moderate-to-severe depression, but those with no or mild anxiety had significantly higher perpetration than those with moderate-to-severe anxiety. There was no statistically significant difference in victimization among different anxiety or depression levels alone (all *p*-values ≥ 0.05). This is among the first studies to examine reported levels of bullying perpetration and victimization among adolescents experiencing anxiety and depression. The findings help to identify adolescents who experience moderate-to-severe levels of depression as an at-risk group for bullying perpetration, who should therefore be a focus of bullying intervention work.

## 1. Introduction

Bullying has been characterized as a major public health problem among adolescents internationally [1,2]. Commonly defined as intentional and repetitious interpersonal aggression that is characterized by an imbalance of power between the perpetrator and victim [3,4], bullying can consist of acts of physical or verbal aggression, social harassment or rejection, and isolation [5,6]. Bullying is a pervasive problem that can have harmful and enduring consequences [7,8]. For example, studies have identified that bullying victimization and perpetration among adolescents can cause a variety of adverse health outcomes. These include physical injuries, social and emotional distress, diminished psychological well-being, increased alcohol and drug use, higher criminality and deviant behaviors, and higher likelihoods of suicidal ideations, attempts, and completions during childhood and throughout a person’s lifespan [5,9,10,11].

Internationally, estimates suggest that 20–33% of school-aged adolescents experience bullying victimization or perpetration [7,12]. For example, Han and colleagues (2017), utilizing nationally representative survey data collected in 2016 from seven provinces across China, reported prevalence rates of 26.10% and 9.03% for bullying victimization and perpetration, respectively, among 3675 school-aged children [5]. According to Benedict and colleagues (2015), “Understanding the risk profile of childhood bullies is essential in gaining a better grasp of this public health problem and in creating useful and appropriate resources and interventions to decrease bullying” (p. 2). Further, given that bullying victimization and perpetration can be a substantial contributor to deleterious health conditions, it is important to identify those most at risk [13]. It is known that certain socio-demographic groups are more likely to be victims or perpetrators of bullying than others [8]. As such, researchers have examined differential rates of bullying perpetration and victimization among a variety of sociodemographic groups in an effort to understand what groups may exude magnified risk profiles for bullying [8,12]. Some of the sociodemographic groups that have exhibited higher rates of bullying include adolescents who live in one-parent or no-parent households [12], adolescents with disabilities [13,14], adolescents who experience adverse childhood experiences [15], or those who are overweight or obese [16]. This line of inquiry has helped to identify specific sub-groups of adolescents who are most in need of attention in prevention efforts to reduce the risk of bullying victimization and perpetration.

Another sociodemographic group that may be at risk for elevated rates of bullying victimization but has not been a major focus in the extant literature are adolescents who experience mental health issues such as anxiety or depression. Anxiety, characterized by excessive fear or worry, and depression, characterized by persistent sad or irrational mood, are considered among the most commonly occurring mental health disorders that develop during adolescence [17,18]. Those who experience anxiety and depression may exhibit behavioral tendencies during the school day that may identify them as being “different” or “weak”, and therefore may fit the mold of those who are typically dominated by others in bullying relations [19]. Similarly, adolescents with anxiety or depression may have difficulties navigating social situations with their peers, which may lead to escalated risk for involvement in bullying victimization [20]. They may also struggle with communicating feelings or emotions, which can heighten their likelihood of bullying others as a way of expressing themselves or responding to others [21]. Taken together, these behavioral and social differences, as well as the high rates of anxiety and depression among adolescents internationally, warrant further examination of bullying victimization and perpetration rates among adolescents experiencing these mental health conditions.

To date, most research exploring the association between psychological health indices and bullying assume anxiety and depression as an outcome of bullying, rather than an antecedent to bullying behaviors [5,11]. However, some evidence exists that suggests that this may be a bi-directional relationship, where those with anxiety and depression may be more likely to engage in bullying perpetration or victimization as well [9]. For example, in an analysis of the data from 63,997 children aged 6 to 17 years in the U.S. from the 2007 National Survey of Children’s Health, it was found that those with a depression diagnosis were 3.31 times more likely to be a bully than those without this diagnosis [9]. The current study will extend the existing literature by including adolescents that experience both anxiety and depression, considering bullying perpetration and victimization, and centering our research in an Eastern province of China. As noted by Han and colleagues [5], while bullying has universal patterns, it also has culturally sensitive characteristics, and understanding bullying in collectivism-oriented countries like China can contribute to our understanding of school violence and bullying in this field, which is generally dominated by studies conducted in individualism-oriented countries such as the U.S. Additionally, adolescents in China typically face high academic stress which is linked to their mental health condition [22], however, it remains unknown whether their mental health conditions (e.g., anxiety and depression) impact their reported levels of bullying perpetration and victimization. As such, the purpose of this study was to examine if adolescents who experience anxiety or depression have higher levels of bullying victimization or perpetration than those who do not. Based on the existing research, we hypothesized that those who experienced moderate or severe depression and anxiety would have higher levels of bullying victimization and perpetration when compared to those who experienced mild or no depression.

## 2. Materials and Methods

### 2.1. Study Design and Participants

We used an observational design for this study. A convenience sample of high-school adolescents from a large local school in the Eastern region of China acted as the participants. The study design and protocols were reviewed and approved by the local school and the corresponding author’s institutional review board. At the time of the data collection, the total number of students in the school was about 4500. The researchers first sought parent/guardian consent for participants younger than 18 years old, and then participant assent from all adolescents before collecting data. The researcher coordinated with local schoolteachers to collect data in person through self-reported surveys during November and December of 2019. The survey was piloted with a sample of 30 students who were not included in the study for readability and completion time. Overall, the response rate was 40.3%, and we received 1816 consent forms and surveys from the students who voluntarily agreed to participate in the study. The final sample included 1481 adolescents who provided complete data for each of the study variables.

### 2.2. Instrument and Measures

#### 2.2.1. Demographic Questionnaire

We collected the participants’ demographic variables, including their age, ethnicity, sex, and the highest education their parents had completed. This demographic information was gathered through a series of four questions. The question on age gathered the participant’s chronological age in number of years. The participants were asked to identify whether they were born as male or female. The ethnicity question asked participants whether they identified as Han or as a minority group. For the question related to parent education background, the participants indicated the highest degree their parents had completed, with options including (a) middle- to high-school diploma, (b) associate or four-year degree, and (c) graduate school or post-baccalaureate degree.

#### 2.2.2. Bullying Perpetration and Victimization

Bullying variables were measured using subscales from the Illinois Bully Scale [23]. The scale asks participants to recall how many times the described bullying perpetration or victimization activities happened in the last 30 days. Specifically, the perpetration subscale contains nine items and the victim subscale has four items. One example perpetration item reads “I upset other students for the fun of it,” and an example victimization item reads “Other students picked on me.” Participants have the option to select one of five responses: “never = 0,” “1–2 times = 1,” “3 or 4 times = 2,” “5 or 6 times = 3,” and “7 or more times = 4.” The aggregated item scores for each subscale indicate the level of bullying perpetration and victimization, where a higher score indicates higher perpetration and victimization, respectively. The aggregated scores do not represent the number of bullying perpetration or victimization instances that the participants had.

#### 2.2.3. Mental Health—Anxiety and Depression

General anxiety was measured using the Chinese version of the General Anxiety Disorder-7 scale (GAD-7). The GAD-7 has seven statements with each presenting a four-point Likert-type scale with choices ranging from 0 = “not at all” to 3 = “nearly every day” for each of the statements. The leading statement for GAD-7 asks, “How often have you been bothered by the following over the past two weeks?” An example of the seven statements from GAD-7 reads “Not being able to stop or control worrying?” The aggregated total scores of the items indicate the level of anxiety, with cut-off scores of 5, 10, and 15 as the levels of mild, moderate, and severe anxiety, respectively [24]. In previous research GAD-7 was shown to possess good concurrent validity (72% sensitivity for social anxiety disorder) and good test–retest reliability (ρ = 0.83) [24].

We used the Patient Health Questionnaire (PHQ-9) to measure the participants’ depression level. PHQ-9 contains nine statements marked with a four-point Likert-type scale [25]. For each of the nine statements, four response options are available, with 0 = “not at all” to 3 = “nearly every day.” The leading question for PHQ-9 asks, “How often have you been bothered by the following over the past two weeks?” For instance, one question reads “Little interest or pleasure in doing things?” The aggregated total scores indicate the level of depression, with cut-off scores of 9, 14, and 20 indicating mild, moderate-to-severe, and severe levels of depression, respectively. In a previous study, PHQ-9 displayed good concurrent validity with an 88% sensitivity for major depression [25]. For this study, we used cutoff scores of 10 and 14 for GAD-7 and PHQ-9 to categorize the participants into “mild or no” and “moderate-to-severe anxiety/depression”, respectively. The measures of bullying perpetration and victimization [23,26], anxiety [24], and depression [25] are available through these respective cited sources.

### 2.3. Data Analysis

We used three types of statistical analyses for this study. Specifically, we used descriptive statistical analyses such as frequency analysis to examine the percentage of different groups/categories of gender, ethnicity, and levels of anxiety and depression. We also computed and reported the arithmetic means and standard deviations of participant age, perpetration, and victimization. Second, we conducted correlation analyses on the continuous variables of participant age, anxiety, depression, perpetration, and victimization, which would help illuminate their linear relationship and help determine the approach for inferential statistical analyses. Finally, we conducted a multivariate analysis of covariance (MANCOVA) to identify the potential differences in participant perpetration and victimization between those with mild–moderate and severe anxiety, and between those with no or mild and those with moderate–severe depression, controlling for participant age, gender, ethnicity, and parents’ highest education. For this analysis, as described in the instrument and measures section, we categorized anxiety and depression into no or mild and moderate-to-severe levels to preserve statistical power and remain consistent with earlier studies [17,18]. Follow-up pairwise comparison (LSD) was also conducted to identify specific differences among the groups. We analyzed the data using IBM SPSS (version 27, Armonk, NY, USA), with α = 0.05 for statistical significance tests.

## 3. Results

The participants ranged in age from 14 to 19, with a mean age of 16.67 years old. Consistent with the student population, a majority of the participants were ethnically Han, and only about 2.5% identified as being members of minority groups. The sample was gender-balanced, with about half (49.3%) being females. As shown in Table 1, only 7.2% of the adolescents reported graduate school or post-baccalaureate degree for their parents’ highest education, 46.9% reported associate or four-year degree, and 45.9% reported middle- or high-school diploma as their parents’ highest education.

Based on the GAD-7 and PHQ-9 data, as shown in Table 1, about 7.1% of the adolescents reported moderate-to-severe depression and 15.2% of them reported moderate-to-severe anxiety, respectively. Anxiety level was highly positively correlated with depression (r = 0.79). As shown in Table 2, bullying perpetration was positively correlated with anxiety (r = 0.16) and depression (r = 0.21) levels, and similarly, victimization was positively correlated with anxiety (r = 0.18) and depression (r = 0.20) levels. Bullying perpetration and victimization were moderately positively correlated (r = 0.50).

Controlling for participant age, gender, ethnicity, and parents’ highest education, adolescent mental health status, anxiety level as measured through GAD-7 (no or mild anxiety vs. moderate or severe; F_21,472_ = 3.34, Pillai’s Λ = 0.01, partial η^2^ = 0.01, *p* = 0.04), and depression level as measured through PHQ-9 (no or mild depression vs. moderate-to-severe; F_21,472_ = 9.34, Pillai’s Λ = 0.01, partial η^2^ = 0.02, *p* < 0.01), had statistically significant impacts on bullying perpetration and victimization both independently and through their interaction (F_21,472_ = 8.99, Pillai’s Λ = 0.01, partial η^2^ = 0.02, *p* < 0.01). As shown in the top section of Table 3, the follow-up pairwise comparison indicated that adolescents with no or mild depression had significantly lower bullying perpetration (2.85, 95% CI = 2.47–3.22) than those with moderate-to-severe depression (5.45, 95% CI = 4.33–6.57), but those with no or mild anxiety had significantly higher perpetration (4.77, 95% CI = 3.73–5.81) than those with moderate or severe anxiety (3.52, 95% CI = 2.95–4.09). There was no statistically significant difference in victimization among different anxiety or depression levels alone (all *p*-values ≥ 0.05). As displayed in the bottom section of Table 3, those with no or mild depression and anxiety showed the lowest levels of perpetration (2.20, 95% CI = 1.96–2.43) and victimization (1.36, 95% CI = 1.21–1.51), which were lower than those with no or mild depression but with moderate-to-severe anxiety regarding perpetration (3.50, 95% CI = 2.78–4.21) and victimization (2.37, 95% CI = 1.91–2.83), respectively (all *p*-values < 0.05). As shown in Table 3, adolescents with moderate-to-severe depression and no or mild anxiety showed the highest level of perpetration (7.35, 95% CI = 5.28–9.41), significantly higher than those with moderate-to-severe depression and anxiety (3.54, 95% CI = 2.67–4.42).

## 4. Discussion

Experiences with bullying victimization and perpetration can have harmful and enduring consequences, and therefore it is important to identify adolescents most at risk for participation in bullying activities [13]. The purpose of this study was to examine if one under-researched group, those who experience anxiety or depression, have higher self-reported levels of bullying victimization or perpetration than those who do not. In our sample, approximately 15.2% and 7.1% of the participants experienced moderate-to-severe anxiety and depression, respectively. These figures are well-aligned with prevalence rates presented in data internationally [17,18], as well as those focused on Chinese adolescents [27].

Unsurprisingly, anxiety and depression levels were each independently positively correlated with both bullying perpetration and victimization. However, several interesting findings emerged during pairwise comparisons when controlling for participant age, gender, ethnicity, and parents’ highest education. Contrary to our hypothesis, no significant differences were found in the levels of bullying victimization when comparing adolescents with no or mild depression to those with moderate-to-severe depression, or adolescents with no to mild anxiety to those with moderate-to-severe anxiety. As such, it appears that adolescents who experience anxiety and depression may not fit the mold of those who are typically dominated in hierarchical bullying relations [19], and therefore may not have magnified risk profiles for bullying victimization. This is a novel finding, as this is the first attempt to explore rates of bullying victimization among persons who experience depression and anxiety, and may have important implications. For example, this finding suggests that the postulated bi-directional relationship between psychological health indices and bullying victimization may be unfounded. That is, although research suggests that adolescents may experience anxiety and depression as a consequence of bullying victimization [5,11], the opposite (that those experiencing high levels of anxiety and depression might experience high levels of bullying victimization) is not supported in this study. This finding should be further examined through longitudinal studies as the current study is limited by its cross-sectional observation.

Unlike bullying victimization, adolescents with no or mild depression had significantly lower rates of bullying perpetration than those with moderate-to-severe depression. This finding is well-aligned with a previous study that demonstrated higher rates of bullying perpetration among adolescents with a diagnosis of depression in the U.S. [9], and extends this finding to Chinese adolescents. Similarly, this finding may support assertions that those who struggle with challenges associated with communicating feelings or emotions, or navigating social situations (e.g., those who experience moderate-to-severe depression), may be at an elevated risk of bullying perpetration as a means of expressing themselves or responding to others [20,21]. Conflictingly, however, pairwise comparisons also revealed that adolescents with no to mild anxiety had significantly higher rates of bullying perpetration than those with moderate-to-severe depression. This finding is surprising, as prior work has demonstrated the inverse relationships among U.S. adolescents [9]. This novel finding diverts from what we may think of persons who experience moderate-to-severe anxiety, who perhaps favor social withdrawal or isolation [28] over the overt aggressive behavior that characterizes bullying perpetration [3,4]. These findings were further supported in pairwise comparisons that took both anxiety and depression into account, where those with moderate-to-severe depression and mild or no anxiety engaged in significantly higher levels of bullying perpetration than any other combinations of groups. Taken together, these findings suggest that when considering interventions to reduce the levels of bullying perpetration among adolescents, creating targeted and specific programs that support adolescents who experience high levels of depression may be particularly warranted.

Several limitations should be considered when interpreting the results of this study. First, being dependent on self-report, responses may have been prone to recall bias due to social desirability. This may be particularly relevant when discussing sensitive topics, such as experiences or levels of bullying, anxiety, and depression. In addition, the participants were not given a specific definition or example of bullying while completing the questionnaire, and analyses did not distinguish between types of bullying (e.g., physical, emotional) or the number of bullying instances. Therefore, further analyses would be required to gain a fuller understanding of the types, frequency, and severity of bullying perpetration and victimization behaviors among adolescents experiencing mental health issues such as anxiety and depression. Additionally, while the sample was representative of the local student population, the low percentage of minority students (2.5%) prevented us from examining the levels of bullying perpetration and victimization these disadvantaged groups face. As such, future research would benefit from over-sampling minority students and examining the levels of bullying perpetration and victimization among those experiencing mental health issues.

## 5. Conclusions

This is among the first studies, to the authors’ knowledge, to examine the levels of bullying perpetration and victimization among adolescents experiencing anxiety and depression. Consistent with our hypothesis and findings from prior literature [9], this study found that adolescents who experienced moderate-to-severe levels of depression engaged in significantly higher levels of bullying perpetration than those who experienced no or mild levels of depression. Conflictingly though, those with moderate-to-severe levels of anxiety engaged in significantly lower levels of bullying perpetration than those with no or mild levels of anxiety, and no significant differences were found among groups with regard to bullying victimization. The findings could help identify adolescents who experience moderate-to-severe levels of depression as an at-risk group for bullying perpetration, who therefore should be the focus of bullying intervention work.

## Figures and Tables

**Table 1:** Participant demographic, anxiety and depression categories.

Age Range	14–19 Years Old (n = 1481)
Gender (%, 95% CI)	
Female	49.3% (46.8–51.9%)
Male	50.7% (48.1–53.1%)
Ethnicity (%, 95% CI)	
Han	97.5% (96.7–98.2%)
Minority	2.5% (1.8–3.3%)
Parent Highest Education (%, 95% CI)	
Middle- or high-school diploma	45.9% (43.5–48.3%)
Associate or four-year degree	46.9% (44.7–49.2%)
Graduate school	7.2% (5.9–8.4%)
Anxiety (%, 95% CI)	
Mild or no anxiety	84.8% (83.0–86.5%)
Moderate-or-severe anxiety	15.2% (13.6–17.0%)
Depression (%, 95% CI)	
Mild or no depression	92.9% (91.7–94.2%)
Moderate-to-severe depression	7.1% (5.9–8.3%)

CI: confidence interval.

**Table 2:** Mean, standard deviation and correlation of bullying, victimization and mental health.

Variable	Mean ± SD	1. Age	2. Perpetration	3. Victimization	4. Anxiety
1. Age (years)	16.67 ± 0.69	1			
2. Perpetration	2.53 ± 4.41	0.10 *	1		
3. Victimization	1.62 ± 2.89	0.07 *	0.50 *	1	
4. Anxiety	6.19 ± 5.23	−0.03	0.16 *	0.18 *	1
5. Depression	5.11 ± 4.95	−0.03	0.21 *	0.20 *	0.79 *

Note: * *p* < 0.01.

**Table 3:** Estimated marginal means of bullying perpetration and victimization among different mental health groups.

Variable	Depression	Anxiety	Mean ^†^	95% CI	*p*
Perpetration	No or mild	—	2.85 *	2.47–3.22	<0.01
	Moderate-to-severe	—	5.45	4.33–6.57	
	—	No or mild	4.77 *	3.73–5.81	0.04
	—	Moderate-to-severe	3.52	2.95–4.09	
Victimization	No or mild	—	1.86	1.62–2.10	0.05
	Moderate-to-severe	—	2.62	1.90–3.34	
	—	No or mild	2.18	1.51–2.85	0.75
	—	Moderate-to-severe	2.30	1.94–2.67	
Perpetration	No or mild	No or mild	2.20 *	1.96–2.43	0.02
		Moderate-to-severe	3.50	2.78–4.21	
	Moderate-to-severe	Mild or no	7.35 *	5.28–9.41	<0.01
		Moderate-to-severe	3.54	2.67–4.42	
Victimization	No or mild	No or mild	1.36 *	1.21–1.51	0.04
		Moderate-to-severe	2.37	1.91–2.83	
	Moderate-to-severe	Mild or no	3.00	1.68–4.33	0.23
		Moderate-to-severe	2.24	1.68–2.80	

Note: * *p* < 0.05 for pairwise comparison; ^†^ estimated marginal means adjusted for participant age, gender, ethnicity, and parents’ education background; CI = confidence interval.

## Data Availability

The data are not publicly available due to local research ethics regulations at the corresponding author’s institution.
